# The role of procalcitonin in reducing antibiotics across the surgical pathway

**DOI:** 10.1186/s13017-021-00357-0

**Published:** 2021-03-24

**Authors:** Massimo Sartelli, Luca Ansaloni, Michele Bartoletti, Fausto Catena, Maurizio Cardi, Francesco Cortese, Francesco Di Marzo, Federico Pea, Mario Plebani, Gian Maria Rossolini, Gabriele Sganga, Bruno Viaggi, Pierluigi Viale

**Affiliations:** 1 Department of Surgery, Macerata Hospital, Macerata, Italy; 2 Surgical Unit 1, University of Pavia, Pavia, Italy; 3 Department of Medical and Surgical Sciences, University of Bologna, Bologna, Italy; 4 Infectious Disease Unit, S. Orsola-Malpighi University Hospital, Bologna, Italy; 5 Department of Emergency Surgery, Parma Maggiore Hospital, Parma, Italy; 6 Department of Surgery, “P. Valdoni” Sapienza University, Rome, Italy; 7 Department of Emergency Surgery, San Filippo Neri Hospital, Roma, Italy; 8 Department of Surgery, Sansepolcro Hospital, Arezzo, Italy; 9 Department of Medical and Surgical Sciences, University of Bologna, Bologna, Italy; 10 S. Orsola-Malpighi University Hospital, Bologna, Italy; 11 Department of Medicine, Laboratory Medicine, University of Padova, Padova, Italy; 12 Department of Experimental and Clinical Medicine, University of Florence, Florence, Italy; 13 Clinical Microbiology and Virology Unit, Florence Careggi University Hospital, Florence, Italy; 14 Emergency Surgery and Trauma, Fondazione Policlinico Universitario “A. Gemelli” IRCCS, Università Cattolica del Sacro Cuore, Rome, Italy; 15 Neurointensive Care Unit, Department of Anesthesiology, Careggi University Hospital, Florence, Italy

**Keywords:** Procalcitonin, Surgical infections, Acute pancreatitis

## Abstract

Procalcitonin (PCT) is widely considered as a highly sensitive biomarker of bacterial infection, offering general and emergency surgeons a key tool in the management of surgical infections. A multidisciplinary task force of experts met in Bologna, Italy, on April 4, 2019, to clarify the key issues in the use of PCT across the surgical pathway. The panelists presented the statements developed for each of the main questions regarding the use of PCT across the surgical pathway. An agreement on the statements was reached by the Delphi method, and this document reports the executive summary of the final recommendations approved by the expert panel.

## Background

Procalcitonin (PCT) is widely regarded as a highly sensitive biomarker of bacterial infection, offering acute care surgeons a key tool in the management of surgical infections. Antibiotics (AB) can be lifesaving when treating bacterial infections but are often used inappropriately, specifically when unnecessary or when administered for excessive durations, and it has been reported that up to 30–50% of the AB given to hospitalized patients may be unnecessary [1]. Treatment courses commonly exceed recommended durations or are targeted toward colonizing or contaminating microorganisms. AB over-prescription has fueled the emergence of antimicrobial resistance as well as unnecessary drug adverse events and propagation of *Clostridioides* difficile infections [1]. This global public health threat has stimulated International and national calls for hospitals to improve AB prescribing practices and implement stewardship programs.

Biomarkers of bacterial infection are attractive because they can provide objective data to increase clinicians’ intuition in starting, withholding, or stopping an AB course.

Among the various biomarkers, PCT has been the most extensively studied.

Procalcitonin, a 116 amino acid polypeptide prohormone of calcitonin, has emerged as a highly sensitive biomarker to help in the diagnosis of bacterial infections. It is primarily synthesized by the thyroid gland C cells, and to a lesser extent by the neuroendocrine tissue of other organs such as the lungs and gastrointestinal tract, and normal PCT levels in the blood are very low. However, its production can be stimulated in almost every organ by inflammatory cytokines and especially bacterial endotoxins, causing high amounts of PCT to be released in the blood. This allows PCT levels to be used as a biomarker of severe inflammation, infection, and sepsis. The higher is the level of PCT, the greater is the likelihood of systemic infection [2].

While there is much evidence that PCT-guided AB treatment in ICU patients with infection and sepsis patient’s results in improved survival and decrease AB treatment duration [3], there is still little evidence on the role PCT-guided treatment in the specific setting of the general and emergency surgery.

## Methods

To clarify the key issues in the use of PCT across the surgical pathway, a multidisciplinary task force of experts met in Bologna, Italy, on April 4, 2019. During the expert board, the panelists presented the statements developed for each of the main questions regarding the use of PCT across the surgical pathway, to reach an agreement on the statements by the Delphi method. Statements reaching an agreement ≥ 80% were approved. The expert panel then met via email to prepare and revise the consensus paper resulting from the meeting, and the manuscript was successively reviewed by all members and ultimately revised as the present manuscript. The review process has been delayed due to the Covid-19 pandemic.

This paper reports the document of the executive summary of the final statements approved by the expert panel.

### What is the role of PCT in guiding AB therapy in patients undergoing surgery for secondary peritonitis?

The post-operative PCT value and its trend, in addition to the evaluation of the patient's clinical condition, can guide the duration of the AB treatment in patients who underwent surgery for secondary peritonitis.

Peritonitis is an inflammation of the peritoneum, and depending on the underlying pathology, it can be infectious or sterile. Infectious peritonitis is divided in primary, secondary, and tertiary peritonitis [1]. Primary peritonitis is a diffuse bacterial infection (usually caused by a single organism) without loss of integrity of the gastrointestinal tract, typically seen in cirrhotic patients with ascites or in patients with a peritoneal dialysis catheter. It has a low incidence in surgical wards and is usually managed conservatively. Secondary peritonitis, the most common form of peritonitis, is an acute peritoneal infection resulting from loss of integrity of the gastrointestinal tract. Tertiary or ongoing peritonitis is a recurrent infection of the peritoneal cavity that occurs > 48 h after apparently successful and adequate surgical source control of a secondary peritonitis. It is more common among critically ill or immunocompromised patients and may be often associated with multidrug-resistant organisms. It is typically associated with high morbidity and mortality. Tertiary peritonitis has been accepted as a distinct entity, even if it is an evolution and a complication of secondary peritonitis.

Intravenous AB treatment is a key component of the first-line management of patients with secondary acute peritonitis. A reasonable and appropriate use of AB is particularly important both to optimize clinical care and to reduce selection pressure on resistant pathogens. Duration of the treatment should be shortened as much as possible as it has been demonstrated that once the source control is established, short courses are as effective as longer courses [4]. The prospective trial by Sawyer et al. demonstrated that in patients with acute peritonitis who underwent an adequate source control, the outcomes after approximately 4 days fixed-duration AB treatment were similar to those after a longer course of AB extended after the resolution of physiological abnormalities [5].

Patients with ongoing signs of peritonitis or systemic illness beyond 5 to 7 days of AB treatment normally warrant a diagnostic investigation to determine whether additional surgery is necessary to address an ongoing uncontrolled source of infection or AB treatment failure. The prolonged and inappropriate use of ABs appears to be a key factor in the rapid rise of antimicrobial resistance worldwide over the past decade [4].

PCT may be useful to guide duration and/or cessation of AB therapy in acute peritonitis. Three studies showed that a PCT-based algorithm could decrease AB exposure in surgical patients. Huang at al [6]. published in 2014 a prospective study investigating whether a PCT-based algorithm could safely reduce AB exposure in patients with acute peritonitis undergoing surgery. PCT levels were obtained pre-operatively, on post-operative days 1, 3, 5, and 7, and on subsequent days if needed. AB were discontinued if PCT was < 1.0 ng/L or decreased by 80% versus day 1, with resolution of clinical signs. The PCT algorithm significantly improved time to AB discontinuation (*p* < 0.001, log-rank test), as the median duration of AB treatment in the PCT group was 3.4 days (interquartile range [IQR] 2.2 days), versus 6.1 days (IQR 3.2 days) in the control group. In 2015, Maseda et al. [7] published a retrospective study including 121 consecutive patients with secondary peritonitis, controlled infection source, requiring surgery, and at least 48-h SICU admission. Treatment was shorter in the PCT-guided group (5.1 ± 2.1 vs. 10.2 ± 3.7 days, *p* < .001), without differences between patients with and without septic shock. PCT guidance produced a 50% reduction in AB duration (*p* < .001, log-rank test). In 2017, Slieker et al. [8] published another study to investigate whether PCT levels could tailor postoperative AB therapy in patients operated for peritonitis. In the subgroup of patients with peritonitis due to gastrointestinal perforation, the authors reported that duration of AB treatment was 7 days in the PCT group versus 10 days in the control group (*p*: 0.065).

### What is the role of PCT in guiding the decision of a relaparotomy in patients undergoing surgery for secondary peritonitis?

The post-operative PCT value and its trend, in addition to the evaluation of the patient’s clinical condition, can guide the decision of a relaparotomy in patients with suspected persistent peritonitis, who already underwent surgery for secondary peritonitis.

In some cases, peritoneal infection can lead to an excessive immune response and sepsis may progress to organ dysfunction syndrome [9]. These patients are severely distressed and may likely experience increased complications and mortality rates leading to a tertiary or ongoing peritonitis, and would benefit from aggressive surgical treatment with successive follow-up surgeries (“re-operations”) to better control organ failure triggered by the ongoing peritonitis [9].The surgical treatment strategies following an initial emergency laparotomy may include a relaparotomy, only when the patient’s condition demands it (“relaparotomy on-demand”), or a planned relaparotomy after 36–48 h with a temporary abdominal closure or an open abdomen.

In 2007, van Ruler et al. [10] published a randomized clinical study comparing planned and on-demand relaparotomy strategies for patients with severe peritonitis.

In this trial, a total of 232 patients with severe intra-abdominal infections were randomized in 2 groups (116 in the planned and 116 in the on-demand). In the planned relaparotomy group, procedures were performed every 36 to 48 h following the index laparotomy to inspect, drain, wash, and perform other necessary abdominal interventions to manage residual peritonitis or new infectious focuses. In the on-demand relaparotomy group, procedures were only performed for patients who showed clinical deterioration or lack of improvement likely due to persistent intra-abdominal pathology. Patients in the on-demand relaparotomy group did not show a significantly lower rate of adverse outcomes compared to patients in the planned relaparotomy group, but they showed a substantial decrease in subsequent relaparotomies and overall healthcare costs. As a consequence, on-demand relaparotomy is recommended as a general strategy in patients with secondary peritonitis [9].

The decision of whether to perform an on-demand relaparotomy in case of a secondary peritonitis is largely subjective and based on personal professional experience. Factors suggesting a progressive or persistent organ failure during early post-operative follow-up analysis are the best indicators of ongoing infection.

Over the past years, PCT was also investigated as a laboratory variable to select patients for on-demand relaparotomy. In 2009, a study by Novotny et al. [11] evaluated PCT as a parameter for early detection of progressing sepsis after surgery of the infective source. PCT ratio appeared to be a valuable aid in deciding if further relaparotomies were necessary after initial operative treatment of an intra-abdominal septic focus. In 2016, the same surgical group published a second prospective study [12]. PCT serum levels were monitored in 234 surgical patients with secondary peritonitis. The PCT ratio on postoperative days 1 and 2 (focus index; FI) was calculated and correlated with the success of the operation. A cutoff value of 1.1 was calculated for the FI. Values below 1.1 indicated poor control of the focus and values above 1.1 correlated with effective treatment. The optimal time for first PCT sampling was found to be 12–24 h after the index operation. After the respective data cleanup, successful elimination of the intra-abdominal focus could be confirmed, with a sensitivity of 93 % and a specificity of 71%.

### What is the role of PCT in identifying patients with uncomplicated diverticulitis who can avoid AB treatment?

Admission PCT value and its trend during the first 2 days after acute diverticulitis onset is useful (in association with clinical evaluation and CT scan findings) in differentiating complicated from uncomplicated disease, avoiding AB treatment.

Diverticular disease of the colon is a common condition affecting up to 60% of individuals older than 60 years [13]. Only 4% of patients will develop acute diverticulitis [14]. Among those, 85% of patients will develop an uncomplicated acute diverticulitis [15]. The definition of uncomplicated acute diverticulitis is often vague and poorly defined. Uncomplicated acute diverticulitis is defined as localized diverticular inflammation without any abscess or perforation. Complicated diverticulitis is usually defined as Hinchey > Ib [16].

The benefit of AB treatment in acute uncomplicated acute diverticulitis has been a point of controversy. In the last few years, several studies demonstrated that antimicrobial treatment was not superior to holding AB therapy in patients with mild uncomplicated diverticulitis, in terms of clinical resolution [17]. Biomarkers can be useful to differentiate complicated from uncomplicated diverticulitis and safely guide AB therapy [18], focusing on a global framework of overuse and the resultant emergence of multidrug-resistant organisms as a strong threat to the welfare of humanity in the twenty-first century [19, 20].

Available reports on biomarkers in diverticulitis have been reviewed recently [21]. C-reactive protein (CRP) is the most commonly evaluated marker in small cohort studies, and shows only moderate sensitivity and specificity. Recent data indicate a role for fecal calprotectin or matrix metalloproteinase in detecting diverticular disease, but it has not yet been clarified whether they are useful in discriminate between complicated and uncomplicated courses of disease [21].

Even if PCT did not show superiority compared to other biomarkers in diagnosing abdominal infections such as acute appendicitis [22], it could have its role in acute diverticulitis [23], differentiating complicated cases, which leads to peritonitis, and that can benefit from AB treatment, from uncomplicated cases that would not benefit from AB treatment. The role of PCT has been assessed by Jeger et al. [18] where most patients with uncomplicated diverticulitis were treated with ABs. This prospective diagnostic cohort study of 115 patients in Switzerland investigated whether PCT could differentiate between complicated and uncomplicated cases of diverticulitis, comparing the results to the gold-standard abdominal computed tomography (CT) scan with the aim to propose a more restrictive use of AB therapy in cases of uncomplicated diverticulitis with appropriate PCT cut-off levels. PCT levels were obtained at admission (day 1), with two pairs of blood cultures, and at day 2. CT scans of the abdomen were performed on physician’s decision and reviewed by radiologists, blinded to the PCT value. All patients were classified as uncomplicated diverticulitis (Hinchey score 0–Ia) or complicated diverticulitis (abscess formation, perforation, peritonitis; Hinchey score Ib–IV). Thirty-five (30%) had a complicated diverticulitis. The median procalcitonin value for uncomplicated diverticulitis was significantly lower compared to complicated diverticulitis (median 0.05, interquartile range [IQR] 0.05–0.06 ng/L vs. median 0.13, IQR 0.05–0.23 ng/L; *p* < 0.0001). In the ROC analysis, the sensitivity and specificity were 81% and 91% when the highest procalcitonin value (days 1 and 2) was considered, with a cut-off value of 0.1 ng/L. CRP and leucocyte count were not significantly different at time of admission.PCT was able to differentiate with a high sensitivity and specificity between complicated and uncomplicated cases of diverticulitis when combined with abdominal CT scans.

As most clinicians still treat uncomplicated diverticulitis with ABs, PCT could be an interesting parameter for guiding treatment and decreasing AB usage [24].

### What is the role of PCT in identifying patients with necrotic pancreatitis needing AB therapy?

Increased PCT level is associated with infection in acute pancreatitis and could guide the AB treatment (avoiding misuse).

Acute pancreatitis (AP) can be mild, moderate, or severe. While mild pancreatitis is commonly self-limited, severe pancreatitis can be associated with the development of complications such as parenchymal/peripancreatic fluid collections and necrosis [25]. Severe AP is defined by single or multiple organ failure lasting more than 48 h, and is associated with a mortality rate as high as 25% [26, 27]. Acute necrotizing pancreatitis is diagnosed when more than 30% of the gland is affected by necrosis and accounts for 5% to 10% of pancreatitis cases [28]. In the subset of patients with organ failure (severe disease) or infected necrosis the mortality rate reaches 30%. The Revised Atlanta classification is used to classify pancreatic fluid collections that develop following AP [26], within the pancreatic parenchyma, adjacent to it, or both; they can be sterile or infected [29]. While sterile necrosis is associated with 5% to 10% mortality rate, it increases to 20–30% when necrosis becomes infected [30–32].

AB treatment in AP has been widely investigated [33], even if the disease itself is not a formal indication for AB therapy [26, 34]. Current guidelines do not recommend prophylactic ABs for the prevention of infectious complications in AP (IAP/APA guidelines, Grade 1B) [35] and American College of Gastroenterology with strong recommendation, moderate quality of evidence [36]. If a source of infection is detected, empiric administration of ABs is justified [37]. It is possible to calculate the rate of ABs use in patients with AP: pancreatic infection occurs in 5% of the patients [38]. Adding a reported 14% to 37.4% extra pancreatic indications (such as cholangitis or pneumonia) [39–41], this would justify a rate of ABs use between 20 and 40% of patients with AP. Hungarian Pancreatic Study Group (HPSG) found that 77.1% of the total study population (600) received AB therapy and 2/3 without signs of infection, only on a preventive basis [41].

The percentage of patients treated with prophylactic ABs varies in population-based studies across the world from 14% in Portugal [42], to 25.5% in Canada [43], 27–58% in the USA [44], 30.7% in the UK [26], 81.4% in India [45], 44.6–69.3% [46], and 74.3% in Japan [47, 48].

Several reasons could stand behind ABs overuse worldwide:

1. Guidelines fail to offer indication for proper AB therapy [42]
2. Misuse of inflammatory biomarkers, as CRP and its influence on prescribing prophylactic ABs [42]
3. Non-adherence to guidelines [33]
4. Defensive medical prescriptions [49–51]

Qu et al. [52] reported the results of a randomized clinical trial stating that PCT-guided (> 0.5 ng/ml) AB regimen is superior over a 2-week prophylactic AB treatment in severe AP. In the study group (35 patients), the duration of AB therapy, intensive care treatment, and length of stay were significantly shorter than in the control group (36 patients) (10.89 ± 2.85 versus 16.06 ± 2.48 days, *p* < 0.001, and 16.66 ± 4.02 days versus 23.81 ± 7.56 days, *p* < 0.001). Any possibility of clinical interpretation of over infection on AP is absolutely strategic [53–55]. Parniczky et al. [56] published a comprehensive paper on the role of ABs in AP, questioning the usefulness of infection’s markers. Their systematic review showed that increased PCT levels but not of CRP, WBC, lipase, or amylase, was associated with infection in the early phase of AP. Neither CRP nor WBC showed differences between patients having positive blood culture versus patients having negative blood culture, suggesting that CRP and WBC have no association with infection at the early phase of AP. However, PCT level showed correlation with infection with acceptable sensitivity and specificity (AUC:0.73).

Furthermore, patients with necrosis have no benefits from AB therapy; it is the organ/s failure along with the infection to increase mortality, not the necrosis itself.

The death rate can increase from 2 to 35% due to bacterial infection of the necrotic pancreatic tissue [41, 53–55]. Organ failure alone was associated with a mortality of 19.8% [51–55], whereas infected necrosis without organ failure has low mortality [53]. In the consensus statements, based on the systematic reviews and retrospective and prospective data analysis, the authors from 62 centers of 23 countries accepted the following statements and recommendations as amendments to the current guidelines [56]:

Statement 1: There is a general overuse of ABs in AP; therefore, centers should make a strong effort to reduce it to a justifiable level (GRADE 1C: strong suggestion, low quality evidence, full agreement).

Statement 2: CRP and WBC values are not associated with infection in the early phase of AP; therefore, CRP and WBC should not be used as biomarkers for decision making concerning AB therapy in the early phase of AP (GRADE 1C: strong suggestion, low quality evidence, full agreement).

Statement 3: Progressive elevation of CRP is part of the inflammatory response in AP; therefore, an upward trend of CRP levels should not be an indicator for AB treatment in the early phase of AP (GRADE 1C: strong suggestion, low quality evidence, full agreement).

Statement 4: Elevation of PCT levels during the early phase of AP is associated with infection; therefore, it can guide the choice to start AB treatment in the absence of proven infection (GRADE 2C: weak suggestion, low quality evidence, full agreement).

Use and misuse of ABs in acute pancreatitis present a global challenge. White blood cell, CRP, lipase, and amylase levels showed no association with infection in the early phase of acute pancreatitis, while PCT levels proved to be a better biomarker of early infection. The use of PCT rather than WBC and CRP could aid physician’s decision-making process, leads to rapid suspicion of infection, and reduces unjustified AB treatment.

### What is the role of PCT in identifying trauma patients needing ABs?

Elevated PCT values during the first 2 days after trauma are more likely to be indicative of trauma impact than of an ongoing status of sepsis because multiple events such as surgery, massive transfusion, and intensive care therapy might influence the PCT concentration.

A long-lasting elevated concentration of PCT in the post-traumatic period, or its repeated increase, is a good marker of developing complications of sepsis.

Mediators of inflammation have been postulated as playing a key role in being responsible for life-threatening complications of multiple trauma patients. PCT is a useful biomarker for diagnosing (bacterial) sepsis; however, elevated PCT in the first days after trauma may be observed in conditions other than sepsis because multiple events such as surgery, massive transfusion, and intensive care therapy might influence its concentration.

A recent literature review on PCT use in trauma-acute care surgery in adult patients performed by Parli et al. showed the importance of this biomarker in identifying infection in trauma and post-operative acute care surgery but no standard approach was recommended [57]. In a prospective study including hospitalized patients with multiple trauma, serum PCT levels were measured in 45 patients: in the 24th hour of observation, a statistically significant increase of PCT concentration versus initial levels were recorded in all patients, then PCT levels decreased significantly whereas PCT concentrations in patients who had complications or died were significantly greater [58]. Liu et al. [59] analyzed blood samples of 30 children with acute trauma: the patients with sepsis showed higher levels of PCT than those with and without systemic inflammatory response syndrome (SIRS) and the healthy controls (*p* < 0.05): the peak level of PCT was observed on day 2 after trauma with a positive correlation with trauma severity acting as an independent predictor for post-trauma sepsis and SIRS. In another prospective study in 94 patients with consecutive trauma > or = 16 years who were admitted to the ICU for an expected stay of > 24 h, patients with trauma presented an early and significant increase in PCT at the moment of septic complications compared with concentrations measured 1 day before the diagnosis of sepsis: 0.85 vs. 3.32 ng/ml for PCT (*p* < 0.001) and 135 vs. 175 mg/L for CRP (*p* = ns) [60]. In another Japanese retrospective series of 75 patients with ISS > 16, the multivariate logistic regression analysis showed that packed red blood cell (PRBC) transfusion was the only independent risk factor for a higher PCT levels on day 1 (*p* = 0.04) confirming that PCT was not a useful infection biomarker in the first days after trauma [61]. A larger retrospective series including 405 patients demonstrated that trauma leads to increased PCT plasma levels dependent on the severity of injury, with peak values on days 1 and 3 (*p* < .05) and a continuous decrease within 21 days after trauma: however, the highest PCT plasma concentrations early after injury were observed in patients with sepsis (6.9 ± 2.5 ng/L; day 1) or severe MODS (5.7 ± 2.2 ng/L; day 1) with a sustained increase (*p* < .05) for 14 days compared with patients with an uneventful posttraumatic course (1.1 ± 0.2 ng/L); moreover, these increased PCT plasma levels during the first 3 days after trauma predicted (*p* < .0001; logistic regression analysis) severe SIRS, sepsis, and MODS [62]. In an US prospective clinical study, PCT was measured from 74 patients with multiple injuries: PCT significantly increased during the first two posttraumatic days in patients with severe multiple injuries (*n* = 24, day 1: 3.37 ± 0.92 ng/L; day 2: 3.27 ± 0.97 ng/L) as compared with patients with identical Injury Severity Score but without abdominal injury (day 1: 0.6 ± 0.18 ng/L; 0.61 ± 0.21 ng/L) [62]. Maier et al. enrolled 73 adult trauma patients admitted to the intensive care unit in a prospective case study: they noted that PCT increased only moderately in most patients and peaked at days 1–2 after trauma, the concentrations rapidly declining thereafter: complications such as sepsis, infection, blood transfusion, prolonged intensive care unit treatment, and poor outcome were more frequent in patients with initially high PCT (> 1 ng/L), whereas increases of CRP showed no positive correlation. These researchers concluded that in patients with multiple trauma due to an accident, the PCT level provides more information than the CRP level since only moderate amounts of PCT are induced, and higher concentrations correlate with more severe trauma and a higher frequency of various complications, including sepsis and infection [63]. In another interesting prospective study, two different groups of patients were studied: one with acute trauma but no clinical evidence of sepsis and the other with clinical evidence of sepsis. Patients with high initial PCT levels (> 2 ng/ml) in severe trauma cases had poor outcomes and risk of developing complications: the difference in PCT levels between days 1 and 4 in group two patients was statistically significant (*p* = 0.006) [64]. In a US Trauma Center, 102 patients were analyzed in 1 year: mean PCT levels were higher for patients with sepsis versus systemic inflammation response syndrome (SIRS) (*p* < 0.0001). Moreover, subjects with PCT values of 5 ng/L or higher showed an increased mortality when compared with those with a PCT of less than 5 ng/L in a univariate analysis (OR, 3.65; 95%,CI 1.03–12.9; *p* = 0.04); in the multivariate logistic analysis, PCT was found to be the only significant predictor for sepsis (odds ratio, 2.37; 95% confidence interval, 1.23–4.61, *p* = 0.01) [65, 66]. This evidence is also confirmed in acute traumatic spinal cord injury patients as showed by Nie et al. who collected 339 cases: 26 (7.7%) of 339 subjects experienced postoperative infectious complication. Patients with infection showed significantly higher PCT levels compared with non-infection (both *p* < 0.01): multivariate logistic regression analysis showed that PCT and CRP levels were independent predicators for postoperative infection. The area under the receiver operating characteristics curve (ROC) of PCT and CRP were 0.82 (95% CI, 0.74–0.91) and 0.68 (95%CI, 0.57–0.78), respectively. A PCT cutoff of 0.1 ng/L had a reasonable sensitivity of 92% to exclude an infection and ABs can be initially withheld: however, in patients with PCT level above 0.5 ng/L, a rapid initiation of ABs may be warranted [67]. Haasper et al. reported during 1 year 94 patients with an injury severity score (ISS) of 16: PCT was better than IL-6 in predicting the development of sepsis showing significant higher plasma levels in group with sepsis from the first day after trauma [68]. In another study, 113 adult multiple-trauma patients admitted to ICU in the first 24 h after trauma were included. Mean PCT and CRP levels were both significantly higher on day 7 compared to day 1 and the final assessment day in patients with an ISS > 20. PCT levels were significantly higher in cases with sepsis, severe sepsis, or septic shock compared with cases who developed SIRS; however, CRP levels were significantly higher only in cases with severe sepsis or septic shock, but not in cases with sepsis alone. These data supported the observation that PCT levels may be a better indicator than CRP levels in the early diagnosis of septic complications in patients with multiple trauma [69]. Another large series grouping a total of 1757 consecutive trauma patients with an ISS > 16 admitted over a 10-year period showed elevated early serum PCT on days 1 to 5 after trauma strongly associated with the subsequent development of sepsis (*p* < 0.01) but not with non-septic infections. The kinetics of IL-6 was similar to those of PCT but did differentiate between infected and non-infected patients after day 5 [70]. In another case control study, PCT, CRP, and IL-6 levels in serum of patients at admission (T1), 12 h after admission (T2), 3 days after admission (T3), and on day 7 (T4) were studied: the serum CRP at the T4 time period was significantly lower than both the T1 and T2 time periods (*p* < 0.05). There were differences in serum PCT, CRP, and IL-6 between the good prognosis and the poor prognosis group at the time of T1–T4 (*p* < 0.05). The expression levels of PCT, CRP, and IL-6 in the serum of patients with poor prognosis were higher than those with good prognosis (*p* < 0.05) [71].

### What is the role of PCT in predicting anastomotic leaks after a colorectal resection?

The post-operative PCT value following colorectal cancer resection can positively identify patients at low risk of anastomotic leakage.

Anastomotic leakage (AL) is the most serious complication of colorectal resection, resulting in increased morbidity and mortality [72]. Incidence varies from 3 to 10% depending on patient characteristics and type of operation [73, 74]. The early diagnosis and treatment of AL, in a latent preclinical phase, are the keys to improve outcomes. However, anastomotic leak may be difficult to diagnose in a preclinical phase and is often recognized in the late postoperative period, when the patient presents with sepsis and peritonitis, therefore increasing associated morbidity and mortality.

In recent years, the enhanced recovery after surgery (ERAS) has spread to the surgical departments around the world for the management of the colorectal cancer patient. It is aimed to optimize perioperative management in order to shorten the hospital stay [75]. Early discharge benefits the patient and reduces healthcare cost; however, it carries a potential risk of developing AL when a patient is already out of the hospital. Delayed presentation and delayed diagnosis of AL can have devastating consequences on the patients. Any reliable marker to guide safe early discharge can be a game changer in the day practice of early discharges. Both CRP and PCT levels following colorectal cancer resection can positively identify patients at low risk of anastomotic leakage [72–74, 76].

### What is the role of PCT in critical acute bacterial skin and skin structures infections (ABSSSI) needing antibiotics?

Critical ABSSSI such as necrotizing soft tissue infections (NSTIs) represent a clinical challenge from the arising of the clinical picture with a mortality rate of 10–30%, septic shock of 30% and long hospital stay(> 6 weeks) [77, 78]. The therapeutic mantra of this acute pathology is “time is tissue” [79]. A rapid diagnosis and combined surgical-antibiotic treatment (< 12 h) are the right way [80] to a complete healing with no extensive tissues damage. PCT plays a pivotal role in any time of the care.

1. Diagnosis and clinical staging (local/systemic)
2. Monitoring in therapeutic response (adequate source control/antimicrobial therapy empiric/targeted)
3. De-escalation
4. Stopping antibiotic therapy

Diagnostic staging of the critical ABSSSI (CABSSSIs) is fundamental in the therapeutic algorithms [81] with an undiagnosed rate at clinical examination rounding 71%. Serological reports associated to TC and/or MR represent the first essential moment in this pathway whit LRINEC seeming obsolete in a modern clinical approach so as CRP as single biomarker. PCT, with or without cytokines, have today a first line role in clinical assessment of the CABSSSIs. The clinical response to the first treatment is fundamental in programming further surgical treatments either in terms of medications under anesthesia or in terms of reoperation for necrosectomy and debridement. Surgical reevaluation is a good rule in operatory room not over 24 h despite the clinical, local, and systemic conditions. During this period, PCT ratio 1^st^/2^nd^ day is a simple and very useful in term of sensibility tool to evaluate this evolution [82]. The Antimicrobial Stewardship policy considers PCT the only useful biomarker in any de-escalation protocol for antimicrobial therapy. Cytokines and other biomarkers are not worldwide used and applied [83].

## Conclusions

PCT is widely regarded as a highly sensitive biomarker of bacterial infection, offering general and emergency surgeons a key tool to aid in the management of surgical infections.

## Data Availability

Not applicable.

## Abbreviations

PLT: Procalcitonin
CRP: C reactive protein
AB: Antibiotic
